# Sporotrichosis: An Emerging Neglected Opportunistic Infection in HIV-Infected Patients in Rio de Janeiro, Brazil

**DOI:** 10.1371/journal.pntd.0003110

**Published:** 2014-08-28

**Authors:** Dayvison Francis Saraiva Freitas, Antonio Carlos Francesconi do Valle, Margarete Bernardo Tavares da Silva, Dayse Pereira Campos, Marcelo Rosandiski Lyra, Rogerio Valls de Souza, Valdiléa Gonçalves Veloso, Rosely Maria Zancopé-Oliveira, Francisco Inácio Bastos, Maria Clara Gutierrez Galhardo

**Affiliations:** 1 Instituto de Pesquisa Clínica Evandro Chagas, Fundação Oswaldo Cruz, Rio de Janeiro, Rio de Janeiro, Brazil; 2 Instituto de Comunicação e Informação Científica e Tecnológica em Saúde, Fundação Oswaldo Cruz, Rio de Janeiro, Rio de Janeiro, Brazil; University of California San Diego School of Medicine, United States of America

## Abstract

Sporotrichosis associated with zoonotic transmission remains a relevant public health problem in Rio de Janeiro, Brazil, affecting a large at-risk population, which includes HIV-infected individuals. We assessed patients co-infected by *Sporothrix* spp. and HIV over time in the context of an unabated sporotrichosis epidemic.

A retrospective cohort retrieved information from a National reference institute for infectious diseases regarding 48 patients with sporotrichosis-HIV co-infection (group 1) as well as 3,570 patients with sporotrichosis (group 2), from 1987 through March 2013. Most patients from group 1 were male (68.8%), whereas women were predominant in group 2 (69.1%; p<0.0001). Patients from group 1 were younger than those from group 2 (μ = 38.38±10.17 vs. 46.34±15.85; p<0.001) and differed from group 2 in terms of their race/ethnic background, with 70.8% non-white patients in group 1 vs. 38.6% from group 2 (p<0.0001). Close to half (∼44%) of the patients from group 1 were hospitalized due to sporotrichosis over time, whereas hospitalization was very unlikely in group 2, among whom approximately 1% were hospitalized over time. Dissemination of sporotrichosis was the main cause of hospitalization in both groups, although it was more common among hospitalized patients from group 1 (19/21 [90.5%] vs. 16/37 [43.2%]; p<0.001). Over the period under analysis, eight patients died due to sporotrichosis (3/48 vs. 5/3,570). The diagnosis of sporotrichosis elicited HIV testing and subsequent diagnosis in 19/48 patients, whereas 23/48 patients were simultaneously diagnosed with the two infections.

HIV infection aggravates sporotrichosis, with a higher incidence of severe disseminated cases and a higher number of hospitalizations and deaths. Underserved populations, among whom sporotrichosis has been propagated, have been affected by different transmissible (e.g., HIV) and non-transmissible diseases. These populations should be targeted by community development programs and entitled to integrated management and care of their superimposed burdens.

## Author Summary

Sporotrichosis is a subcutaneous, worldwide-distributed mycosis, endemic in some areas and is caused by dimorphic fungi from the complex *Sporothrix schenckii*. Its association with zoonotic transmission remains a relevant public health problem in Rio de Janeiro, Brazil, affecting a large at-risk population, which includes HIV-infected individuals. A comprehensive search of a National reference institute for infectious diseases' database retrieved information regarding 48 patients with sporotrichosis-HIV co-infection (group 1), as well as 3,570 patients with sporotrichosis (group 2), registered from 1987 through March 2013. Group 1 mainly comprised young, non-white men, while group 2 was predominantly comprised of white middle-aged women. HIV infection aggravates sporotrichosis, as seen with patients from group 1, who presented more severe disseminated sporotrichosis, a higher need for hospitalization and risk of death due to this mycosis. Due to its aggressive presentation, sporotrichosis elicited HIV testing and subsequent diagnosis in 19/48 patients. Underserved populations, among whom sporotrichosis has propagated, have been affected by different transmissible (e.g., HIV) and non-transmissible diseases. These populations should be targeted by community development programs and entitled to integrated management and care of their superimposed burdens.

## Introduction

Sporotrichosis is a subcutaneous mycosis with a worldwide distribution that is endemic in some areas of Latin America. The infection is caused by a dimorphic fungus previously described as a single species, *Sporothrix schenckii* [1], that is now understood as a complex of different species of clinical interest [2]. Molecular studies have identified *Sporothrix globosa*, *Sporothrix mexicana*, *Sporothrix brasiliensis* and *S. schenckii* as responsible for sporotrichosis in different regions [2]–[7]. The classical infection is associated with traumatic subcutaneous inoculation of soil, plants, and organic matter contaminated with fungus, with rare cases of transmission from infected animals [1]. Most patients with sporotrichosis have a localized disease limited to the skin and subcutaneous tissue (lymphocutaneous and cutaneous fixed forms), comprising up to 95% of cases. Dissemination to various organs and systems occurs in rare cases, mainly in immunosuppressed individuals [8].

In Rio de Janeiro state, Brazil, sporotrichosis has become an urban endemic/epidemic phenomenon, with transmission from infected cats to humans in ∼91% of human cases [9]. These cases came from the greater metropolitan area of Rio de Janeiro (the capital city of Rio de Janeiro state), forming a sporotrichosis belt. These areas are low-income, underserved areas, with scarce and inadequate health services [9]–[10].

The increase in the number of cases of the disease has been continuous for more than 15 years and remains on the rise, affecting vulnerable groups of humans, as well as domestic and stray cats [10]. In 2012, Freitas et al. [8] described the clinical manifestations and evolution of sporotrichosis in human immunodeficiency virus (HIV)-infected patients in the largest case series reported to date worldwide.

In Brazil, the HIV/AIDS epidemic has been stable and concentrated in some urban areas, mostly affecting men who have sex with men and female sex workers. The overall epidemic dynamics have switched from a population of higher socioeconomic status to individuals from low-middle and lower socioeconomic strata [11]. These dynamics favor a superposition of HIV spread with other infections such as tuberculosis and leprosy, which have been uniquely prevalent in contexts of poverty and pronounced socioeconomic and social geographic inequality [12], [13].

The present study summarizes data from a large dataset of sporotrichosis cases, consisting of 3,570 patients registered from 1987 up to March 2013, as well as 48 patients co-infected by HIV and sporotrichosis, who sought care at a reference infectious disease unit located in Rio de Janeiro, Brazil. Based on our previous clinical data [8] our hypothesis is that patients co-infected by HIV with low CD4^+^ cell count are prone to worse outcomes as sporotrichosis evolves as an opportunistic condition.

## Methods

### Ethics statement

The Research Ethics Committee of the Instituto de Pesquisa Clínica Evandro Chagas (IPEC)/Fundação Oswaldo Cruz (Fiocruz), RJ, Brazil, approved this study under the protocol number 0001.0.009.000-06. All patients involved were anonymized for privacy and ethics purposes.

### Study site

IPEC is a National reference center for infectious diseases. Since the beginning of the sporotrichosis epidemic in Rio de Janeiro in 1998, this center has been the main referral center for the treatment of this mycosis in the state due to its certified laboratory and the optimal infrastructure of its clinical and ancillary services. All services are delivered free of charge, and referral is agile. Patients may be referred to IPEC from any health service (public or private) or may spontaneously seek care. In addition, the AIDS program at IPEC began in 1986 and is currently one of the largest providers of primary, specialty, and tertiary care for HIV-infected individuals and AIDS patients in Rio de Janeiro State.

### Study design

For this retrospective cohort study, a systematic search of IPEC's clinical database was conducted to identify cases of sporotrichosis-HIV co-infection that were registered from 1987 through March 2013, as well as sporotrichosis cases, overall. All patients diagnosed with sporotrichosis confirmed by laboratory tests were included, as well as patients living with HIV under follow-up in the institute's cohort. Patients with sporotrichosis co-infected with HIV (hereafter denominated “group 1”) and patients with sporotrichosis (“group 2”) constituted the groups under analysis. Additional analyses were conducted on patients from the IPEC HIV/AIDS cohort who were diagnosed with the following opportunistic mycoses: histoplasmosis, cryptococcosis and paracoccidioidomycosis. Only patients aged 18 years old or more at the time of registration were included in the study.

### HIV diagnosis

The diagnosis of HIV infection followed Brazilian Ministry of Health regulations, which are summarized as follows: an immune-enzymatic method (ELISA) test plus immune-fluorescence or western blot. HIV serology was conducted in all cases of disseminated cutaneous and disseminated cases of sporotrichosis or evidence of HIV signs or symptoms. A non-paired random HIV test was performed with the stored blood samples of 850 patients from group 2 who were registered from 2000 through 2008.

### Sporotrichosis diagnosis

Isolation of *Sporothrix* spp. from clinical specimens was used as the study's key inclusion criterion, as previously described by Barros et al. [14]. When the patient had clinical or laboratory signs of HIV-related immunodeficiency (CD4^+^ count <200 cells/µL), fungal dissemination was investigated by culturing his/her sputum, blood, urine, and cerebrospinal fluid (CSF) samples, as well as by endoscopic and imaging studies, as previously described by Freitas et al. [8]. Clinical cases of sporotrichosis were classified as localized (lymphocutaneous and fixed forms), cutaneous disseminated and disseminated forms. This last form may involve extracutaneous tissues such as the skeletal system, lungs, testis, nervous system, and mucous membranes [15].

### Data collection

Demographic data included the following categories: gender, age, ethnicity/color, city of residence and education. Ethnicity/color was established as white or non-white (brown [mulatto] and black were grouped together here) by the administrative staff at the time of registration in the institute until the year 2005, after which this information was then self-reported by the patients. The clinical characteristics of sporotrichosis, as well as the associated morbidity and mortality, were summarized by the variables as follows: “date of diagnosis of HIV infection”; “HIV plasma viral load”; “CD4^+^ cell count” and “use of highly active antiretroviral therapy (HAART) for patients from group 1; “date of diagnosis of sporotrichosis”; “hospitalization due to sporotrichosis” (yes/no); main cause for hospitalization among those hospitalized as a consequence of sporotrichosis (dissemination, secondary bacterial infection, hypersensitivity reaction [erythema multiforme or erythema nodosum], and local worsening); comorbidities (cardiovascular disease [ICD-10:I51.6], diabetes [ICD-10:E10–E14], chronic obstructive pulmonary disease (COPD) [ICD-10:J44], and alcoholism [ICD-10:F10]); number and length of hospitalizations due to sporotrichosis; as well as deaths secondary to sporotrichosis.

Additional analyses took into consideration the year of occurrence of opportunistic mycoses (histoplasmosis, cryptococcosis and paracoccidioidomycosis) in patients from the IPEC HIV/AIDS cohort.

Sociodemographic, clinical and laboratory data were entered into contingency tables and cross-compared using parametric and non-parametric tests (e.g., chi-square or Fisher's exact tests for categorical variables, and t-tests or the Wilcoxon-Mann-Whitney test for means for continuous variables). A p-value lower than 0.05 was defined as statistically significant for the sake of our analyses. Analyses were carried out with the help of SPSS (17.0), R (version 2.15.3) and Microsoft Office Excel 2013.

## Results

**Figure 1:**
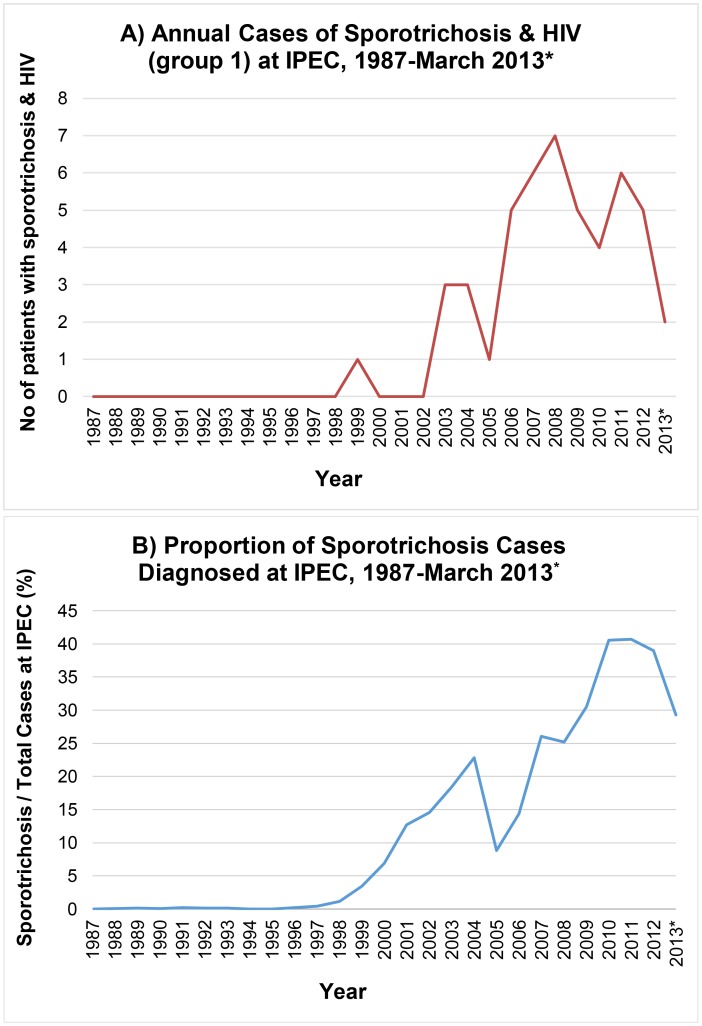
Annual number of patients with sporotrichosis at IPEC from 1987 through March 2013. A) Sporotrichosis and HIV (group 1) and B) annual proportion of patients diagnosed with sporotrichosis among all patients diagnosed at IPEC.

From 1987 through March 2013, 3,618 patients were diagnosed with sporotrichosis at IPEC and 48 of them were co-infected with HIV (Figure 1). The main sociodemographic aspects of the patients with sporotrichosis are summarized in Table 1.

**Table 1:** Demographics of the patients diagnosed with sporotrichosis at IPEC from 1987 through March 2013.

Variable	Category	Group 1	Group 2	p-value
**Sample**		48	3570	
**Gender**	Male	33 (68.8%)	1102 (30.9%)	<0.0001
	Female	15 (31.2%)	2468 (69.1%)	
**Age**	Mean	38.4	46.3	<0.001
**Ethnicity/color**	White	14 (29.2%)	2148 (60.2%)	<0.0001
	Non-white	34 (70.8%)	1380 (38.7%)	
	Unknown	-	42 (1.1%)	
**Place of Residence**	Hyperendemic^1^	46 (95.8%)	3469 (97.2%)	>0.60
****	Non-hyperendemic	2 (4.2%)	101 (2.8%)	
**Education**	0–8 years	28 (58.3%)	1712 (48.0%)	>0.20
	>8 years	19 (39.6%)	1806 (50.6%)	
	Unknown	1 (2.1%)	52 (1.4%)	

1 Hyperendemic: Rio de Janeiro metropolitan region.

When cross-comparing patients from groups 1 and 2, some interesting differences were evident. Individuals from both groups clustered in the same geographic area, i.e., the outskirts and impoverished neighborhoods of the metropolitan region of Rio de Janeiro (95.8% and 97.2%, respectively) and had a similar low educational background. Among the patients from group 1, approximately 40% of patients had more than 8 years of schooling and a slightly higher proportion of patients from group 2 (50.6%) had a similar educational level (this difference was not statistically significant; p>0.2).

Most patients from group 1 were male (68.8%), whereas women predominated in group 2 (69.1%; p<0.0001). Patients from group 1 were younger than those from group 2 (mean age = 38.38±10.17 years vs. 46.34±15.85 years; p<0.001) and differed from those from group 2 in terms of their race/ethnic background, with 70.8% non-whites in group 1 vs. 38.6% from group 2 (p<0.0001).

Sporotrichosis has been associated with some major harms and risks. There were 69 hospitalization events due to sporotrichosis among 58 patients (i.e., some of them were hospitalized more than once; Table 2). However, the proportion of patients who required hospitalizations over time markedly differed between groups. Close to half (∼44%) of the patients from group 1 were hospitalized over time, whereas hospitalization was a very unlikely event among patients from group 2, among whom approximately 1% were hospitalized over time as a consequence of conditions directly or indirectly associated with sporotrichosis. In addition to the fact that they were much more frequent, hospitalizations were longer among patients from group 1 compared to group 2 (37 days vs. 21 days; not statistically significant, as expected for such small figures). Among patients from group 1, the need for hospitalization due to sporotrichosis was 42 times higher than among those from group 2. This difference becomes even more pronounced (greater than 50 times higher) when successive hospitalizations to perform complex or extensive diagnostic and therapeutic procedures, such as parenteral antifungal treatment, supportive therapy and its associated monitoring, are taken into consideration (Table 2).

**Table 2:** Hospitalization of patients with sporotrichosis at IPEC from 1999 through March 2013.

	Group 1	Group 2	p-value
**Sample**	48	3570	
**No of hospitalizations** 1	28	41	<0.0001
**Mean length of hospitalization (days)**	37	21	>0.05
**Hospitalized**	21/48 (43.8%)	37/3570 (1.0%)	<0.0001
**Main cause for hospitalization**			
**Dissemination**	19/21 (90.5%)	16/37 (43.2%)	<0.001
**Other** 2	2/21 (9.5%)	21/37 (56.8%)	<0.001
**Secondary bacterial infection**	1/21 (4.8%)	14/37 (37.8%)	
**Hypersensitivity reaction**	1/21 (4.8%)	5/37 (13.5%)	
**Local worsening**	-	2/37 (5.4%)	
**Comorbidity**	3/21 (14.3%)	20/37 (54.1%)	<0.01
**Cardiovascular**	1/21 (4.8%)	12/37 (32.4%)	<0.05
**Diabetes**	2/21 (9.5%)	4/37 (10.8%)	
**COPD** 3	-	2/37 (5.4%)	
**Alcoholism**	-	2/37 (5.4%)	

1 Four HIV patients had two hospitalizations and one had four hospitalizations, whereas four non-HIV patients had two hospitalizations;

2 p-value for the other causes of hospitalization, grouped (secondary bacterial infection, hypersensitivity reaction, local worsening);

3 COPD: Chronic Obstructive Pulmonary Disease.

Dissemination of sporotrichosis was the main cause for hospitalization in both groups, although it was more common among hospitalized patients from group 1 (19/21 [90.5%] vs. 16/37 [43.2%]; p<0.001). These hospitalized patients from group 1 had a mean CD4^+^ count of 125 cells/µl (range: 7–323 cells/µl), a median viral load of 4,967 copies/ml (range: <50 [detection threshold] - >500,000 copies/ml [values on the right side of the curve were collapsed into this category]), and only 3 were under HAART in a regular basis, while the other 18 patients were not under this specific treatment (not prescribed, not adherent or on HAART for less than 3 months). On the other hand, local and/or hypersensitivity manifestations of sporotrichosis were predominant in hospitalized patients from group 2 (p<0.001). At least one comorbidity was present in 54.1% (20/37) of the hospitalized patients from group 2, but this was a relatively rare event (3/21 [14.3%]) among hospitalized patients from group 1 (p<0.01) (see Table 2). This difference was mainly due to cardiovascular diseases (p<0.05), whereas the prevalence of diabetes was relatively similar in both groups. Three hospitalized patients belonging to group 1 had other opportunistic infections at the time of hospitalization: one patient had pulmonary tuberculosis, another patient had cryptococcal meningitis and another had cytomegalovirus retinitis.

During the period under analysis, eight patients died due to sporotrichosis (3/48 vs. 5/3,570); thus, death attributed to sporotrichosis occurred 45 times more frequently in patients from group 1.

Sporotrichosis elicited HIV testing and subsequent diagnosis, due to its severe clinical presentation, in 19 patients who were unaware of their HIV status. Four other patients were simultaneously diagnosed with the two infections. However, three of them presented localized disease and HIV-related conditions (chronic seborrheic dermatitis, herpes zoster, weight loss and dyspnea) and one had sporotrichosis exclusively, with lymph node involvement a few days after the diagnosis of the HIV infection. In total, 23 patients were simultaneously diagnosed with the two infections. Among the remaining 25 patients, 13 were under follow-up in the HIV/AIDS cohort and 12 were referred by other HIV/AIDS clinical providers.

In the non-paired random HIV testing performed among 850 patients from group 2, 1 sample was positive (0.12%).

In an effort to better understand the role of sporotrichosis vis-à-vis other endemic or classic opportunistic mycoses affecting patients with HIV/AIDS belonging to the IPEC HIV/AIDS cohort, an additional search of the institution's database was performed. It included 5,385 patients living with HIV/AIDS and focused on diagnoses of histoplasmosis, cryptococcosis and paracoccidioidomycosis reported among patients living with HIV/AIDS since 1987 (Table 3, see also Supporting Information S1).

**Table 3:** Periodic diagnosis of opportunistic mycoses in patients with HIV at IPEC from 1987 through March 2013.

Year	Cryptococcosis		Histoplasmosis		Paracoccidioidomycosis		Sporotrichosis	
	No	% Increment^1^	No	% Increment	No	% Increment	No	% Increment
**≤1992**	15	-	10	-	2	-	-	-
**1993–1996**	16	6,67%	16	60,00%	1	−50,00%	-	-
**1997–2000**	5	−68,75%	14	−12,50%	1	0,00%	1	-
**2001–2004**	10	100,00%	15	7,14%	4	300,00%	6	500,00%
**2005–2008**	9	−10,00%	12	−20,00%	4	0,00%	19	216,67%
**2009–2013**	18	100,00%	22	83,33%	7	75,00%	20	5,26%
**Total**	73		89		19		48	

1 Current period/previous period.

In recent years, cases of sporotrichosis have been on the rise, whereas figures for histoplasmosis, cryptococcosis and paracoccidioidomycosis have been low or declining (Table 3).

## Discussion

The first case of sporotrichosis and HIV co-infection diagnosed at IPEC was reported in 1999, roughly coinciding with the emergence of sporotrichosis as a public health issue in Rio de Janeiro [16]. Since then, the increase in the number of patients with this co-infection was roughly proportional to the overall increase in the number of sporotrichosis cases over time, with the exception of 2005. During this year, the specialized outpatient service's staff was dramatically reduced by a combination of factors. This anomaly certainly biased our time series, the clientele's demands that year could not be properly addressed. As of early 2006, the service regained its full working capacity.

A greater than proportional increase in patients with sporotrichosis co-infected with HIV has been documented in recent years. This may be explained by an actual acceleration of the propagation of sporotrichosis in the last seven years, by a comprehensive HIV screening by clinicians aware of the possibility of co-infection and the seriousness of this type of double health burden, or a combination of both factors. A possible bias to be pointed is the fact that the most severe cases are prone to be referred to IPEC even more frequently than the regular cases of sporotrichosis. In the context of the stability of the HIV/AIDS epidemic, it is unlikely that a local outbreak of HIV has been taking place among people with sporotrichosis. The modest prevalence of other fungal co-infections and their decline in recent years speak in favor of a unique pattern followed by sporotrichosis, which may or may not be associated with HIV/AIDS.

According to the norms issued by the Brazilian Ministry of Health, sporotrichosis does not constitute a condition for which provider-initiated testing and counseling for HIV is mandatory or strongly recommended (such as for patients diagnosed with tuberculosis). As the clinical forms of sporotrichosis in HIV-infected patients varied according to the patients' immune status, we might be missing asymptomatic seropositive patients from group 2 who would present benign forms of sporotrichosis (lymphocutaneous and fixed), which correspond to ∼90% of the clinical presentation of sporotrichosis cases among our patients overall [9], [14]. Because of this possibility, we performed a non-paired random HIV testing in approximately one-quarter of the blood samples collected from patients form this group. Despite the limitations intrinsic to this type of strategy (besides the fact that this strategy is the only one that could be accomplished retrospectively), the low prevalence (0.12%) speaks in favor of a modest degree of misclassification (i.e., people assigned to group 2 who actually belong to group 1). Obviously, misclassification constitutes a bias that may compromise any cross-comparative analysis. In this specific study, one tends to overestimate harms and risks associated with co-infection because cases who did not present any evident clinical problem tend to be erroneously included in group 2.

The sociodemographic characteristics of group 1, which is composed of a majority of young males, differ from group 2, which is mostly composed of middle-aged women engaged in domestic duties as previously described in this epidemic [9], [14]. This finding may reflect the dynamics of the HIV/AIDS epidemic in Brazil. Recent studies have shown that the number of affected men is still increasing, especially among young men who have sex with men (MSM). At the end of 2012, the estimated overall prevalence of HIV in Brazil was 0.4% but reached 10.5% among MSM [11].

Most of the patients in group 1 and almost half of the patients from group 2 had eight years or less of education. Neglected diseases are often found in poor, marginalized sections of the population who have restricted access to formal education [17]. Furthermore, non-white ethnicity/color was prevalent only in group 1. This finding could point to a subgroup with worse social and economic conditions, which historically reflects inequality in access to health services that tends to be secondary to multiple partially overlapping factors, such as social status, gender, race/ethnicity, place of residence, etc. [18]. However, a misreporting of this variable cannot be ruled out because in Brazil, there is a large degree of miscegenation and the registered ethnicity/color was based on skin color instead of proxies of genetic ancestry [19].

As previously described, HIV clearly modifies the natural history of sporotrichosis and is associated with a broad spectrum of this mycosis [8]. T CD4^+^ cells have a pivotal role for the control of sporotrichosis [20] and these cells are exactly the main target of HIV infection. As documented by our findings, HIV co-infection was found to be associated with a much higher incidence of severe disseminated cases and a greater number of hospitalizations and deaths. These patients presented a severe immunosuppression and viral replication. Although further experimental evidence is sorely needed, observational data corroborate the underlying reasoning provided by the pathophysiology and immunology of both infections. This increase in severity may represent a serious issue to the public health and to the economy of the region.

Moreover, we should keep in mind that sporotrichosis is not always a benign disease and can lead to hospitalization and death even in patients without immunosuppression. Since July 2013, sporotrichosis was included among the conditions for which a formal report to the State Health Secretariat is mandatory. This change is an auspicious one, which may contribute to much more accurate reports in the near future.

Secondary bacterial infection and hypersensitivity reactions were found to be relevant causes of hospitalization among patients from group 2. In this group, the presence of comorbidities was key in terms of more serious conditions and more frequent hospitalizations. *S. brasiliensis*, the main etiologic agent of this specific epidemic, seems to be more virulent than other species of the *S. schenckii* complex [21] and may cause pronounced hypersensitivity reactions.

It is remarkable that 47.9% of patients were simultaneously diagnosed with the two infections due to the presence of opportunistic sporotrichosis or other HIV-related conditions. It is clear that this subgroup of patients did not have adequate access to the early diagnosis of HIV infection and have entered into HIV care relatively late, which seems to have increased their chance of acquiring additional infections and the risk of dying as a consequence of AIDS in the first year of diagnosis [22].

Unlike sporotrichosis, which has been a sustained and protracted threat in recent years, HAART has been associated with a stabilization of the number of classical opportunistic mycoses, such as histoplasmosis and cryptococcosis [23], as documented in our database.

Sporotrichosis incidence among HIV-infected patients has been increasing on a continuous basis, and at the end of the study period, the incidence was roughly comparable to that of histoplasmosis and cryptococcosis. In contrast, paracoccidioidomycosis does not seem to be associated with HIV infection.

Since the beginning of the sporotrichosis zoonotic epidemic in Rio de Janeiro, IPEC has been the main regional reference center for this mycosis. The same does not apply to the management of classical opportunistic mycoses; therefore, the IPEC figures for sporotrichosis tend to be close to the actual number of cases in the metropolitan area of Rio de Janeiro, but IPEC figures certainly underestimate classical opportunistic mycoses that are usually managed by clinicians and infectious diseases from a large network of primary and secondary HIV/AIDS care locations.

In 2012, the clinical profiles of 21 of these 48 patients of group 1 who were followed up in 1999–2009 were analyzed by our team [8]. A search for international reports in English at that time accounted for 34 cases historically reported. The present study updated this information up to March 2013, making this case series the largest worldwide to the best of our knowledge.

The harms and risks associated with the propagation of sporotrichosis in a disenfranchised population affected by different medical and social conditions are of concern. Among these multiple, partially superimposed burdens, sporotrichosis and HIV co-infection is of great concern. Both infections are preventable and should be targeted by integrated programs. It would be naïve to suppose that these deprived and underserved communities do not face other major overlapping problems, such as substance misuse, crime, and a myriad of other problems associated with inadequate sanitation, waste disposal, access to healthy food, etc. Community development and structural changes fostered by comprehensive public policies and private-public partnerships remain the only real alternative to permit these communities to regain full citizenship and acceptable standards of life. The unabated spread of sporotrichosis in the second largest and most industrialized metropolitan area in Brazil, for more than a decade, is evidence that we are unfortunately far from reaching these goals.

## Supporting Information

Checklist S1 **STROBE checklist.**
(DOC)

Supporting Information S1 Annual occurrence of opportunistic mycoses in HIV-infected patients at IPEC from 1987 through March 2013.*
(TIF)
