# Supplementary material for: Sporotrichosis: An Emerging Neglected Opportunistic Infection in HIV-Infected Patients in Rio de Janeiro, Brazil
Source: PLoS Negl Trop Dis. 2014 Aug 28;8(8):e3110. doi: 10.1371/journal.pntd.0003110 (PMC4148221; doi:10.1371/journal.pntd.0003110)
Supplement: Checklist S1 — STROBE checklist. (DOC) [file pntd.0003110.s001.doc]

STROBE Statement—checklist of items that should be included in reports of observational studies

|  | | Item No | Recommendation |
| --- | --- | --- | --- |
| **Title and abstract** | | 1 | (*a*) Indicate the study’s design with a commonly used term in the title or the abstract  Included in the revised abstract. |
|  |  |  | (*b*) Provide in the abstract an informative and balanced summary of what was done and what was found Done. |
| Introduction | | | |
| Background/rationale | | 2 | Explain the scientific background and rationale for the investigation being reported  Available in the revised Methods section. |
| Objectives | | 3 | State specific objectives, including any prespecified hypotheses  The main hypothesis, besides the overall description of the whole cohort, concerns the outcomes of people with sporotrichosis with and without HIV, as available in the revised manuscript . |
| Methods | | | |
| Study design | | 4 | Present key elements of study design early in the paper  Done. |
| Setting | | 5 | Describe the setting, locations, and relevant dates, including periods of recruitment, exposure, follow-up, and data collection  The study was retrospective, comprising cases reported from 1987-2013 and this information is available in the revised manuscript. |
| Participants | | 6 | (*a*) *Cohort study*—Give the eligibility criteria, and the sources and methods of selection of participants. Describe methods of follow-up Done.  *Case-control study*—Give the eligibility criteria, and the sources and methods of case ascertainment and control selection. Give the rationale for the choice of cases and controls  *Cross-sectional study*—Give the eligibility criteria, and the sources and methods of selection of participants |
|  |  |  | (*b*) *Cohort study*—For matched studies, give matching criteria and number of exposed and unexposed  The study was not a matched study and did not include unexposed patients since all patients have a diagnosis of sporotrichosis. Notwithstanding, patients with and without HIV infection were compared.  *Case-control study*—For matched studies, give matching criteria and the number of controls per case |
| Variables | | 7 | Clearly define all outcomes, exposures, predictors, potential confounders, and effect modifiers. Give diagnostic criteria, if applicable Done. |
| Data sources/ measurement | | 8* | For each variable of interest, give sources of data and details of methods of assessment (measurement). Describe comparability of assessment methods if there is more than one group |
| Bias | | 9 | Describe any efforts to address potential sources of bias  The key biases were mentioned and discussed, such as selection bias which takes place in any clinical centre, as well as the lack of optimal HIV screening, since in Brazil sporotrichosis is NOT a disease eliciting mandatory HIV screening. |
| Study size | | 10 | Explain how the study size was arrived at  The study does NOT comprise a sample, but rather the universe of patients diagnosed and reported in the period under review. In this sense, sample size calculation does not apply. Of course, this may be associated with some limitations we tried to address in the revised manuscript. |
| Quantitative variables | | 11 | Explain how quantitative variables were handled in the analyses. If applicable, describe which groupings were chosen and why Done. |
| Statistical methods | | 12 | (*a*) Describe all statistical methods, including those used to control for confounding  Fully available in the revised Methods section. |
|  |  |  | (*b*) Describe any methods used to examine subgroups and interactions Not applicable. |
|  |  |  | (*c*) Explain how missing data were addressed Done. |
|  |  |  | (*d*) *Cohort study*—If applicable, explain how loss to follow-up was addressed  In a retrospective study, losses had happened over the years and cannot be fully addressed by any means besides retrieving any available information from all databases. This was thoroughly accomplished by our team.  *Case-control study*—If applicable, explain how matching of cases and controls was addressed  *Cross-sectional study*—If applicable, describe analytical methods taking account of sampling strategy |
|  |  |  | (*e*) Describe any sensitivity analyses Not applicable. No other metrics besides standard clinical and laboratory diagnosis, as mandated by the Brazilian Ministry of Health, were used. |
| Results | | | |
| Participants | 13* | (a) Report numbers of individuals at each stage of study—eg numbers potentially eligible, examined for eligibility, confirmed eligible, included in the study, completing follow-up, and analysed Done. | |
|  |  | (b) Give reasons for non-participation at each stage Done. | |
|  |  | (c) Consider use of a flow diagram We think the revised Methods section is clear enough. Anyway, it can be easily designed, in case the editor and referees think it could be helpful. | |
| Descriptive data | 14* | (a) Give characteristics of study participants (eg demographic, clinical, social) and information on exposures and potential confounders Done. | |
|  |  | (b) Indicate number of participants with missing data for each variable of interest Done. | |
|  |  | (c) *Cohort study*—Summarise follow-up time (eg, average and total amount) Done. | |
| Outcome data | 15* | *Cohort study*—Report numbers of outcome events or summary measures over time Done. | |
|  |  | *Case-control study—*Report numbers in each exposure category, or summary measures of exposure | |
|  |  | *Cross-sectional study—*Report numbers of outcome events or summary measures | |
| Main results | 16 | (*a*) Give unadjusted estimates and, if applicable, confounder-adjusted estimates and their precision (eg, 95% confidence interval). Make clear which confounders were adjusted for and why they were included Analyses were straightforward and did not incorporate additional adjustments since they are not applicable to the universe of clinical cases under analysis. | |
|  |  | (*b*) Report category boundaries when continuous variables were categorized Done. | |
|  |  | (*c*) If relevant, consider translating estimates of relative risk into absolute risk for a meaningful time period Not applicable. | |
| Other analyses | 17 | Report other analyses done—eg analyses of subgroups and interactions, and sensitivity analyses | |
| Discussion | | | |
| Key results | 18 | Summarise key results with reference to study objectives Done. | |
| Limitations | 19 | Discuss limitations of the study, taking into account sources of potential bias or imprecision. Discuss both direction and magnitude of any potential bias Done, as mentioned above. | |
| Interpretation | 20 | Give a cautious overall interpretation of results considering objectives, limitations, multiplicity of analyses, results from similar studies, and other relevant evidence  Interpretation of results incorporated all suggestions made by the referee (such as the putative role of diabetes mellitus, the entangled role of HIV replication and CD4+ depletion). | |
| Generalisability | 21 | Discuss the generalisability (external validity) of the study results Generalisability is clearly limited due to the very particular characteristics of the universe of clinical cases demanding a given service. Notwithstanding, data refer to the main reference centre in Rio de Janeiro and correspond to the largest number of cases, worldwide. No other setting, worldwide has been dealing with the continuous spread of sporotrichosis in the context of a service where HIV co-infection is a relevant condition. | |
| Other information | | | |
| Funding | 22 | Give the source of funding and the role of the funders for the present study and, if applicable, for the original study on which the present article is based Done. | |

*Give information separately for cases and controls in case-control studies and, if applicable, for exposed and unexposed groups in cohort and cross-sectional studies.

**Note:** An Explanation and Elaboration article discusses each checklist item and gives methodological background and published examples of transparent reporting. The STROBE checklist is best used in conjunction with this article (freely available on the Web sites of PLoS Medicine at http://www.plosmedicine.org/, Annals of Internal Medicine at http://www.annals.org/, and Epidemiology at http://www.epidem.com/). Information on the STROBE Initiative is available at www.strobe-statement.org.
