# Supplementary figures and images for: Sporotrichosis: An Emerging Neglected Opportunistic Infection in HIV-Infected Patients in Rio de Janeiro, Brazil
Source: PLoS Negl Trop Dis. 2014 Aug 28;8(8):e3110. doi: 10.1371/journal.pntd.0003110 (PMC4148221; doi:10.1371/journal.pntd.0003110)

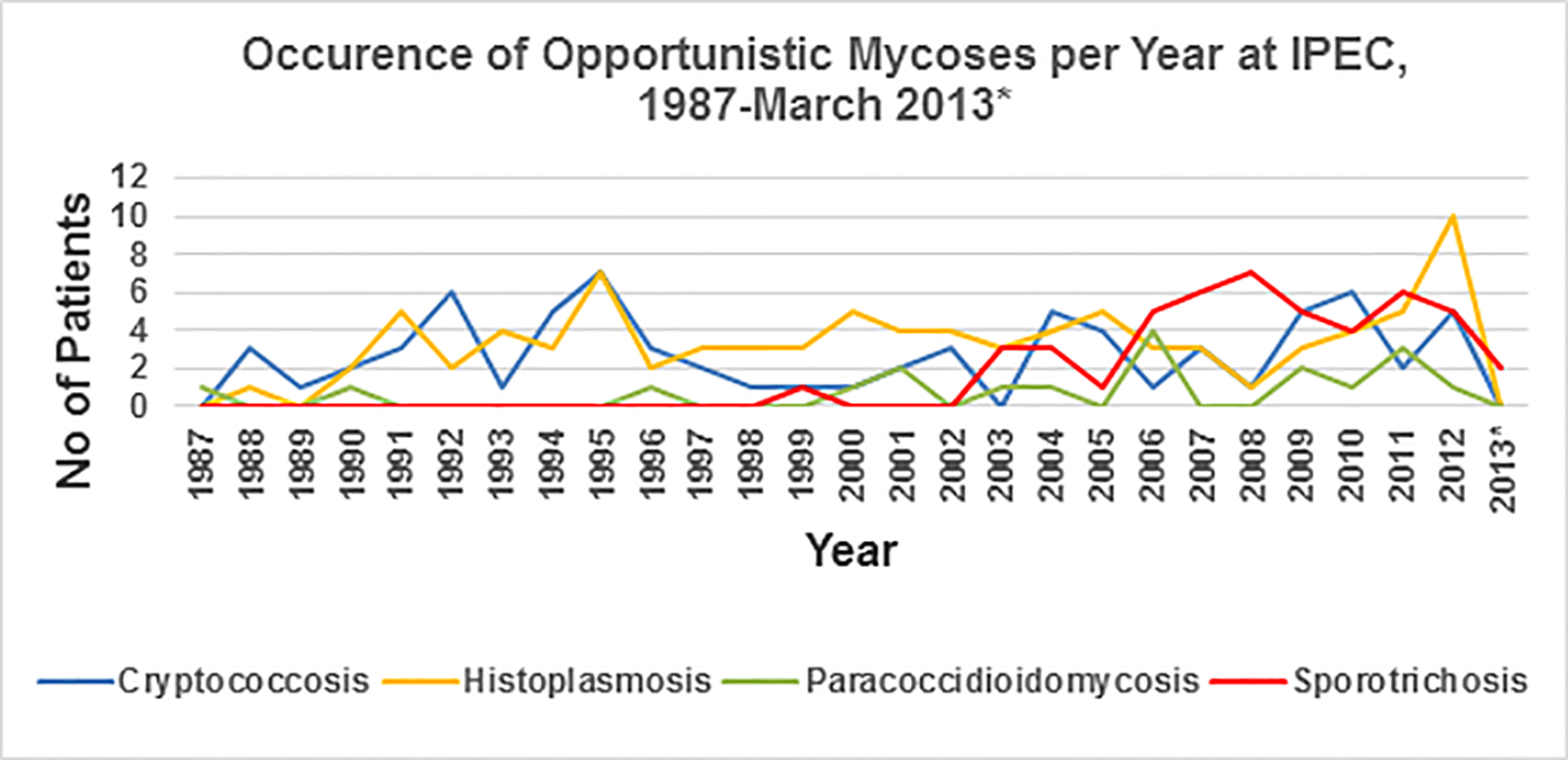

Supplement: Supporting Information S1 — Annual occurrence of opportunistic mycoses in HIV-infected patients at IPEC from 1987 through March 2013.* (TIF) [file pntd.0003110.s002.tif]
